# Expediting rare disease diagnosis: a call to bridge the gap between clinical and functional genomics

**DOI:** 10.1186/s10020-020-00244-5

**Published:** 2020-11-25

**Authors:** Samantha N. Hartin, John C. Means, Joseph T. Alaimo, Scott T. Younger

**Affiliations:** 1 Center for Pediatric Genomic Medicine, Children’s Mercy Kansas City, Kansas City, MO 64108 USA; 2 Children’s Mercy Research Institute, Children’s Mercy Kansas City, Kansas City, MO 64108 USA; 3 Department of Pediatrics, University of Missouri-Kansas City School of Medicine, Kansas City, MO 64110 USA; 4 Department of Pathology and Laboratory Medicine, Children’s Mercy Kansas City, Kansas City, MO 64108 USA; 5 Department of Pediatrics, University of Kansas Medical Center, Kansas City, KS 66160 USA

**Keywords:** Rare disease diagnosis, Functional genomics, Massively parallel genomic assays, Pooled CRISPR screening

## Abstract

Approximately 400 million people throughout the world suffer from a rare disease. Although advances in whole exome and whole genome sequencing have greatly facilitated rare disease diagnosis, overall diagnostic rates remain below 50%. Furthermore, in cases where accurate diagnosis is achieved the process requires an average of 4.8 years. Reducing the time required for disease diagnosis is among the most critical needs of patients impacted by a rare disease. In this perspective we describe current challenges associated with rare disease diagnosis and discuss several cutting-edge functional genomic screening technologies that have the potential to rapidly accelerate the process of distinguishing pathogenic variants that lead to disease.

## Background

Approximately 400 million individuals worldwide are directly affected by a rare disease (Wakap et al. 2019; Global Genes:RARE Facts 2020). Roughly 70% of rare diseases are exclusively pediatric-onset and 30% of children with a rare disease will not live to 5 years of age (Wakap et al. 2019; Global Genes:RARE Facts 2020). At present, the average time from disease onset to accurate diagnosis for a rare disease is 4.8 years (Global Genes:RARE Facts 2020; Blöß et al. 2017). Reducing the time required for disease diagnosis holds the promise of improving the quality of life for rare disease patients and in some cases may provide a window for therapeutic intervention that would otherwise be missed.

Improved DNA sequencing technologies and decreases in the cost of DNA sequencing have led to the routine use of whole exome sequencing (WES) and whole genome sequencing (WGS) in a clinical setting. While the application of these technologies has greatly facilitated the identification of disease-associated genetic variants, the rate of diagnosis for rare disease remains below 50% (Soden et al. 2014; Yang et al. 2014; Lee et al. 2014). Increased accessibility of DNA sequencing has also had a pronounced impact on the field of functional genomics. High-throughput sequencing-based assays have made it possible to simultaneously profile the functional capacity of thousands of DNA sequences in a single experiment (Melnikov et al. 2012; Kheradpour et al. 2013). Despite the inherent power of these experimental approaches, their application in clinical settings have been limited.

Here we propose that high-throughput functional assays capable of profiling the impact of clinically detected genetic variants be implemented directly within clinical genome sequencing centers. We provide an overview of several high-throughput assays, covering details of their technical execution along with the practical limitations of each approach. Importantly, the techniques we describe can be incorporated into most clinical sequencing platforms without the need to modify existing laboratory infrastructure. These powerful genomic technologies have the potential to rapidly accelerate the process of identifying genetic variants, particularly rare variants, that are likely to be pathogenic and could dramatically reduce the amount of time required for rare disease diagnosis.

## The current state of rare disease diagnosis

In the United States a rare disease is defined as a condition that afflicts fewer than 200,000 individuals (Wakap et al. 2019). Those impacted by a rare disease typically harbor extremely rare, often de novo, genetic variants that are not observed in the general population. The clinical application of WES/WGS technologies has been instrumental in improving the ability to detect these rare variants and their use has doubled the number of Mendelian disease gene associations over the course of the last ten years (Fernandez-Marmiesse et al. 2018). When combined with variant interpretation guidelines outlined by the American College of Medical Genetics (ACMG) and the Association for Molecular Pathology (AMP) these technologies routinely achieve diagnostic rates of 25–35% in pediatric cohorts with idiopathic disease(Yang et al. 2014; Iglesias et al. 2014; Farwell et al. 2014; Hartman et al. 2019). Although these diagnostic rates are encouraging, at present the majority of rare disease patients that undergo WES/WGS remain undiagnosed.

Characterizing and categorizing genetic variants identified through WES/WGS is a highly involved and time-consuming process. The ACMG/AMP guidelines recommend that variants be subjected to a comprehensive assessment that incorporates population frequency, computational/predictive algorithms to infer effects of variants on protein function, experimental evidence with in vitro assays directly measuring variant function or animal models mimicking phenotypic features, segregation analysis in multigeneration/offspring pedigrees, variant origin/configuration, and additional information or data from reputable sources (Richards et al. 2015). In many cases there is insufficient data to satisfy these criteria and variants are categorized as variants of uncertain significance (VUS). Large-scale data repositories such as gnomAD and ClinVar that store and curate variant information have greatly assisted variant characterization and reduced the levels of VUS reporting in recent years (Hartman et al. 2019; Landrum et al. 2013, 2015, 2017; Karczewski et al. 2020). However, these reductions have not been accompanied by improved diagnostic rates indicating that alternative approaches to assess variant pathogenicity are needed (Hartman et al. 2019).

Among the most compelling lines of evidence to support variant pathogenicity is the presence of empirical data demonstrating the impact of a given variant on genome function. This data is particularly valuable for noncoding variants as their functional consequences are challenging to predict using existing computational algorithms. However, empirical data for rarely observed genetic variants is often nonexistent. Moreover, the time and costs associated with performing detailed functional studies for a large number of potentially pathogenic variants can be prohibitive. Advances in high-throughput sequencing-based functional screening technologies (e.g. massively parallel genomic assays, large-scale pooled CRISPR screening) over the past several years now provide scalable mechanisms to assign functional properties to large catalogs of variants. These approaches can be used to rapidly distinguish clinically detected variants with an increased likelihood of pathogenicity and facilitate the prioritization of variants that warrant in-depth evaluation.

## Large-scale variant profiling using massively parallel genomic assays

One common experimental approach used to explore the functional consequences of a genetic variant has been the use of plasmid-based reporter assays. These assays can be engineered to harbor specific variant sequences within exons, introns, or even noncoding regulatory regions of a transgenic reporter gene. Individual reporter constructs can be introduced into cultured cells and transgene expression and/or function can be evaluated using relevant methods. In recent years several plasmid-based reporter approaches have been adapted to multiplexed formats that permit the characterization of thousands of genetic variants simultaneously using high-throughput sequencing-based readouts. These massively parallel genomic assays have been utilized to profile published catalogs of disease-associated genetic variants and distinguish variants with functional implications (Tewhey et al. 2016; Cheung et al. 2019). Incorporating these massively parallel genomic assays into clinical workflows has the potential to significantly accelerate the process of pinpointing pathogenic variants.

Massively parallel genomic assays are performed with diverse libraries of reporter constructs that are generated through a combination of array-based DNA synthesis and large-scale molecular cloning. In general, thousands of 100 to 200 nt oligos containing genomic sequences centered around variants-of-interest are designed and synthesized. The resulting oligo pool is cloned directly into a reporter construct that can subsequently be introduced into cultured cells. Several companies specializing in oligonucleotide synthesis offer pooled oligo synthesis as a service and the process of library cloning can be completed within a few days using basic molecular cloning techniques. At the completion of an assay RNA or DNA is harvested and the functional impact of each variant is determined by measuring the relative abundance of individual library elements through targeted sequencing.

**Fig. 1:**
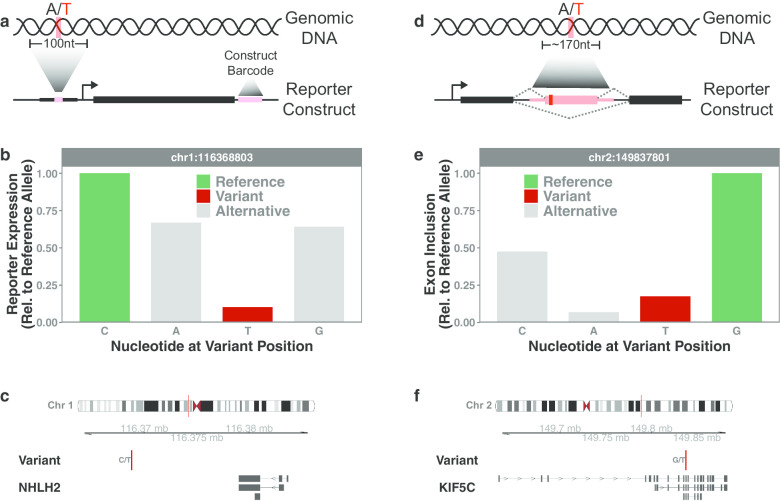
Massively parallel genomic assays for profiling genetic variants. **a** Assay design for profiling the impact of genetic variants on gene expression. **b** Representative experimental results for a rare genetic variant that alters gene expression in a reporter assay. **c** Genomic location of rare variant profiled in (**b**). **d** Assay design for profiling the impact of genetic variants on RNA splicing. **e** Representative experimental results for a rare genetic variant that alters exon inclusion in a splicing assay. **f** Genomic location of rare variant profiled in (**e**)

The vast majority of disease-associated genetic variants occur in noncoding regions of the human genome (Hindorff et al. 2009; Gusev et al. 2014). Noncoding variants located within functional regulatory elements that influence the expression of disease genes can be pathogenic (Spielmann and Mundlos 2016). However, most noncoding variants occur in regions of the genome with no prior functional annotation and predicting their pathogenicity remains a major challenge. Massively parallel genomic assays have provided a powerful platform for profiling the impact of noncoding variants on the regulatory capacity of genomic sequences. Briefly, these assays incorporate variant-containing sequences upstream of a barcoded reporter gene and high-throughput sequencing is used to quantify barcode abundance as a proxy for the regulatory potential of the upstream sequence (Fig. 1a). These expression assays have been used to profile thousands of noncoding variants reported by the 1000 Genomes Project as well as variants identified through various GWAS studies (Tewhey et al. 2016; Ulirsch et al. 2016). We propose that similar expression assays be implemented within clinical sequencing platforms to profile clinically detected variants. In our undiagnosed patient population at Children’s Mercy Kansas City we typically observe several thousand "family-specific" rare variants and hundreds of “patient-specific" (de novo) rare variants per individual, the majority of which occur within noncoding regions of the genome. We’ve functionally profiled thousands of these variants using the approaches described here resulting in the discovery of many variants located in genomic regions proximal to disease-relevant genes that have a significant impact on regulatory activity. Importantly, standard clinical practices would not have prioritized these particular variants for detailed investigation. In Fig. 1b we show representative data for one such rare variant that dramatically alters gene expression in our reporter assays. This variant is located on chromosome 1, roughly 13 kb downstream of NHLH2 (Fig. 1c). The NHLH2 gene encodes a transcription factor that directly regulates expression of Prohormone Convertase 1/3, an enzyme associated with dwarfism in mouse models (Zhu et al. 2002; Fox and Good 2008). In agreement with these models, the clinical features of the patient harboring this particular variant include short stature, macrocephaly, and mesomelic arm/leg shortening.

Genetic variants have also been shown to cause large-effect disruptions in RNA splicing (Cheung et al. 2019). However, most variants that disrupt splicing occur outside of canonical splice sites (Cheung et al. 2019). As a result, these variants are difficult to predict based on sequence alone and their identification requires experimental testing. Several different massively parallel genomic assays have been designed to profile the impact of genetic variants on RNA splicing. These assays typically incorporate variant-containing exons into a fixed intronic region of a reporter gene and high-throughput sequencing is used to evaluate inclusion/exclusion of the exon (Fig. 1d). These splicing assays have been used to profile variants cataloged in the Human Gene Mutation Database as well as variants identified through the Exome Aggregation Consortium (ExAC) (Cheung et al. 2019; Soemedi et al. 2017). Collectively, these studies have identified more than one thousand naturally occurring variants that significantly disrupt RNA splicing. We propose that similar approaches be implemented to profile the effect of clinically detected genetic variants on splicing. Many of the rare variants we’ve identified in our undiagnosed patients at Children’s Mercy Kansas City occur within gene bodies (introns/exons) and are not predicted to impact protein sequence or function. We’ve functionally profiled hundreds of these variants and identified dozens that significantly alter RNA splicing, bringing us a major step closer to determining which of these variants may be pathogenic. In Fig. 1e we show representative data for a rare variant that dramatically alters exon inclusion in our splicing assays. This variant is located on chromosome 2, within an intron of KIF5C (Fig. 1f). Previously reported variants in the KIF5C gene have been associated with intellectual disability and epilepsy (Ligt et al. 2012; Poirier et al. 2013). Consistent with these observations, the clinical features of the patient harboring this particular variant include intellectual disability, seizures, and vision loss.

In contrast to current standard clinical approaches that rely heavily on prior knowledge, the massively parallel genomic assays discussed here provide a mechanism to directly evaluate the functional consequences of variants at the molecular level. However, these assays are plasmid-based and some variants may exhibit distinct functional characteristics when profiled outside of their endogenous genomic context. As a result, we anticipate that a subset of consequential variants may register as false negatives when using these techniques. Although the data generated by these assays might not alone be sufficient to achieve a clinical diagnosis, the information they provide can be used to rapidly prioritize variants for follow-up validation and significantly reduce the time required to illuminate those that are pathogenic. Importantly, the infrastructure and equipment required to perform these assays are already in place within clinical sequencing centers. A typical assay profiling several thousand variants can be completed in less than one month for less than $10 K, including sequencing costs.

Although massively parallel genomic assays have proven to be powerful experimental tools, there are a number of practical limitations that must be considered prior to their implementation. For example, array-based DNA synthesis is currently limited to oligo lengths under 200 nt which constrains the size of genomic regions that can be profiled. In addition, the sequence composition of some genomic regions may preclude DNA synthesis and/or result in biases during the cloning process. Lastly, the genomic background of the cell types in which assays are performed can impact variant function. For this reason, we recommend that assays are performed across a panel of cell lines representing a diversity of cellular contexts.

## High-throughput variant characterization using cell-based phenotypic assays

Many genetic variants may lead to disease through mechanisms that are more complex than disruptions in basic molecular processes (e.g. RNA splicing, direct transcriptional regulation). Distinguishing the functional consequences of these variants may require assays that are capable of profiling cellular phenotypes. Advances in genome editing technology, specifically CRISPR/Cas9, have dramatically improved the ability to engineer cellular models with specific genetic alterations (Cong et al. 2013). Similar to the massively parallel genomic assays described previously, many CRISPR-based approaches have been adapted to multiplexed/pooled formats that permit the functional screening of thousands of genetic perturbations in parallel (Shalem et al. 2014). These methods can be utilized to phenotypically profile the consequences of clinically detected variants in high-throughput and dramatically improve the ability to discern variants that are likely to be pathogenic.

Pooled CRISPR-based screens are lentiviral-delivery genetic assays that introduce a diversity of genetic perturbations into a large cell population (Shalem et al. 2014; Piccioni et al. 2018). Libraries of oligos encoding sgRNAs that target genomic sequences-of-interest are designed and synthesized using array-based DNA synthesis. The resulting oligo pool is cloned into a lentiviral backbone that can be used to generate a complex pool of lentivirus. The pooled library virus is transduced into a large Cas9-expressing cell population at a low multiplicity of infection such that the majority of infected cells harbor a single viral integrant. Following the application of selective pressure (e.g. proliferation, differentiation) genomic DNA is isolated from the remaining cell population and targeted sequencing of the viral cassette is used to quantify sgRNA abundance as a proxy for the functional impact of the genetic perturbation. While pooled CRISPR screening has proven to be a robust technology, it does have practical limitations. For example, large-scale pooled CRISPR screens can require hundreds of millions of cells which may preclude the use of some cellular models. Furthermore, screens that require the isolation of cell populations with complex phenotypes may be prohibitive.

**Fig. 2:**
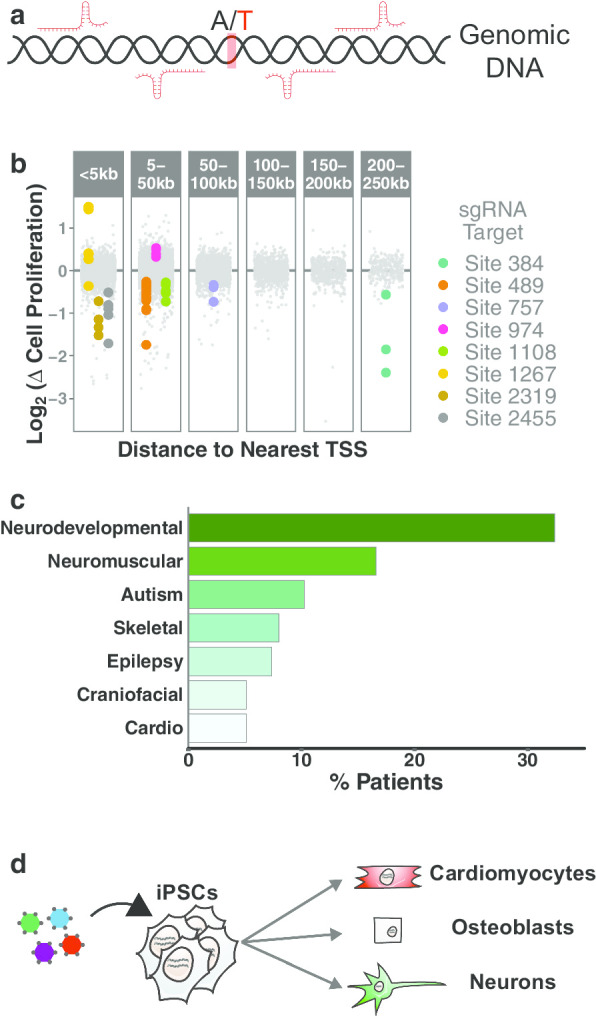
Pooled CRISPR screening of genetic variants.** a** Design of an sgRNA library for screening genetic variants. **b** Representative data from a proliferation-based pooled CRISPR screen targeting putative noncoding regulatory elements. A selection of sgRNAs targeting common genomic sites have been labeled. **c** Frequency of clinical presentations in our undiagnosed patient population. **d** Schematic of pooled CRISPR differentiation screens

Genome-wide pooled CRISPR screens have been widely used to identify genetic dependencies in varied cellular models. The majority of these screens have focused on the knockout (CRISPRko), inhibition (CRISPRi), or activation (CRISPRa) of protein-coding genes (Shalem et al. 2014; Sanson et al. 2018). However, most disease-associated variants occur within noncoding regions of the genome (Gusev et al. 2014; Spielmann and Mundlos 2016). Recently, several reports have described the use of pooled CRISPR screening technologies to identify essential noncoding regulatory elements in the human genome (Korkmaz et al. 2016; Gasperini et al. 2016; Han et al. 2018; Borys and Younger 2020). These approaches have utilized pooled CRISPR libraries that target noncoding genomic regions-of-interest as opposed to protein-coding genes (Fig. 2a). Representative data from one of our CRISPRi-based pooled screens targeting putative noncoding regulatory elements are shown in (Fig. 2b; Borys and Younger 2020). We find that this approach is able to identify regulatory elements > 100 kb from the nearest annotated gene that are essential for cell proliferation. This same strategy can be applied to clinically detected genetic variants by perturbing genomic regions that harbor these variants in cell-based models and evaluating the impact on cellular phenotypes (Fig. 2a). Although this method does not model the exact variants detected in patients, it can be used to illuminate genomic regions that may be functionally implicated in disease etiology.

Most CRISPR screens reported to date have utilized cell-based models that have been highly optimized to reflect specific biological contexts. However, undiagnosed rare diseases are often associated with an array of clinical presentations and generating a cell-based model that can precisely reflect a large number of patients is not feasible. For this reason, we propose the use of generalized cell models that can be screened to identify variant-harboring regions that are associated with frequently observed clinical features. For example, over 30% of the undiagnosed patient population at Children’s Mercy Kansas City have neurodevelopmental challenges, 8% are impacted by skeletal abnormalities, and 5% have cardiovascular complications (Fig. 2c). We have implemented a pooled CRISPRi/CRISPRa screening strategy in which iPSCs are transduced with variant-targeting CRISPR libraries and subsequently differentiated into relevant cell types (e.g. neurons, osteoblasts, cardiomyocytes) (Fig. 2d). The enrichment/depletion of sgRNAs in differentiated cells can be used to pinpoint genomic regions that are likely to be associated with disease-related phenotypes. Once these regions are identified, iPSC lines harboring individual clinically detected variants can be engineered to more precisely model disease biology.

The CRISPR screening approach we describe here is a robust and efficient way to screen thousands of genetic variants for potential roles in disease-related phenotypes. Moreover, the use of CRISPR technology enables the perturbation of variant-containing sites in an endogenous genomic context. As with the massively parallel genomic assays discussed previously, pooled CRISPR screens do not require specialized laboratory equipment and can readily be performed in most clinical sequencing centers. Experiments designed to screen several thousand variants can be completed in less than two months for less than $15 K, including sequencing costs.

Pooled CRISPR screening will be particularly useful for profiling the functional implications of noncoding genetic variants. However, the current state of large-scale CRISPR screening technology is limited to random indel mutations (CRISPRko) or the repression/activation of targeted genomic regions (CRISPRi/CRISPRa). Consequently, these screens do not perfectly model the impact of clinically detected variants. As CRISPR-based screening methods continue to advance it may become possible to functionally screen large numbers of specific variants through pooled format adaptations of precision editing technologies (e.g. base editing, search-and-replace editing) (Shen et al. 2018; Anzalone et al. 2019).

## Conclusions

The ability to rapidly assign experimentally determined functional properties to clinically detected genetic variants will have profound impacts on rare disease diagnosis. In addition to existing resources, clinical geneticists will have access to empirical data that will facilitate more informed decisions related to variant pathogenicity. This information will reduce the time required to analyze individual patient genomes, increase patient throughput, and ultimately translate to improved rates of diagnosis. Moreover, the barriers to generating this data are minimal as many high-throughput functional assays do not require modifications to existing laboratory infrastructure nor do they require patient specimens.

The experimental strategies we discuss here are intended to complement, not replace, current standard practices in variant interpretation. Moreover, the functional assays we have described are mainly suited for Mendelian diseases. Experienced clinical geneticists will always be needed to critique experimental results and to investigate diseases with more complex genetic contributions. Rare disease diagnosis will remain a constant challenge, but bridging the gap between clinical and functional genomics could provide an accelerated path to diagnosis for many rare disease patients that are still searching for answers.

## Data Availability

Not applicable.

## Abbreviations

WES: Whole exome sequencing
WGS: Whole genome sequencing
